# Drug Resistance–Associated Mutations in *Mycoplasma genitalium* in Female Sex Workers, Japan

**DOI:** 10.3201/eid2106.142013

**Published:** 2015-06

**Authors:** Takashi Deguchi, Mitsuru Yasuda, Kengo Horie, Kensaku Seike, Mina Kikuchi, Kohsuke Mizutani, Tomohiro Tsuchiya, Shigeaki Yokoi, Masahiro Nakano, Shinji Hoshina

**Affiliations:** Gifu University, Gifu, Japan (T. Deguchi, M. Yasuda, K. Horie, K. Seike, M. Kikuchi, K. Mizutani, T. Tsuchiya, S. Yokoi, M. Nakano);; Hoshina Clinic, Kyoto, Japan (S. Hoshina)

**Keywords:** Mycoplasma genitalium, 23S rRNA, ParC, female sex worker, bacteria, parasites, Japan

## Abstract

*Mycoplasma genitalium* was detected in 21 (14.1%) of 149 vaginal swab samples and in 1 (0.7%) of 149 throat washing samples from female sex workers during 2013–2014 in Japan. Prevalences of *M. genitalium* with macrolide resistance–associated 23S rRNA mutations and fluoroquinolone resistance–associated *parC* alterations were 47.1% and 36.8%, respectively.

For *Mycoplasma genitalium* infections, azithromycin regimens have been considered first-line treatments, and fluoroquinolone regimens, including those of moxifloxacin and sitafloxacin, have been effective second-line treatments (*1*). However, the proportion of *M. genitalium* harboring macrolide or fluoroquinolone resistance–associated mutations has been increasing in male and female patients with *M. genitalium* infections (*2**–**5*), and treating *M. genitalium* infections with current antimicrobial chemotherapies is increasingly difficult (*5**,**6*).

The prevalence of *M. genitalium* infections in women at low risk for sexually transmitted infections (STIs) is reportedly 2.0%, with the range for most cohorts being <1%–5%. In high-risk populations, the prevalence is 0%–42% (*7*). For female sex workers (FSWs), the range of *M. genitalium* prevalence rates is reportedly 12%–26% (*7**–**10*). FSWs could be a reservoir of *M. genitalium* infections, but little is known about drug resistance in *M. genitalium* in FSWs.

In this study, vaginal swab and throat washing samples collected from 149 FSWs were examined for the presence of *M. genitalium*. Positive specimens were then tested for drug resistance–associated mutations in the *M. genitalium* DNA.

## The Study

This cross-sectional prospective study was approved by the Institutional Review Board of the Graduate School of Medicine, Gifu University, Japan (reference number 22–11). A total of 149 FSWs who attended Hoshina Clinic, Kyoto, Japan, for regular screening for STIs from August 2013 through January 2014 were enrolled in this study after informed consent was obtained. The women were 19–47 years of age (mean 29 years). All performed fellatio on their clients without use of condoms. Six (4.0%) had received antimicrobial drug treatment (i.e., azithromycin, clarithromycin, ceftriaxone, or amoxicillin) for gonococcal or chlamydial infections during the 3 months before visiting the clinic. Sixty-five (43.6%) had histories of STIs, including gonococcal infections, chlamydial infections, genital condyloma, genital herpes, and syphilis. Other sociodemographic information, sexual history, or HIV serologic status was not obtained from most participants. At clinic visits, all were asymptomatic. On genital examination, however, genital herpes was found in 1 (0.7%), and mucopurulent vaginal discharge was found in 3 (2.0%).

Vaginal swab and throat washing samples were collected from all 149 women, as previously recommended (*11*). These specimens were tested by using Cobas 4800 CT/NG (Roche Molecular Systems, Pleasanton, CA, USA) to detect *Chlamydia trachomatis* and *Neisseria gonorrhoeae*. The specimens were also tested for *M. genitalium*, *M. hominis*, *Ureaplasma urealyticum*, and *U. parvum*, as previously recommended (*12*). The DNA specimens were then stored at −80°C. The 1 throat washing positive for *M. genitalium* was tested by PCR with primers specific for the 23S rRNA genes of the genital mycoplasmas, which were used in the PCR-based assay. The PCR product was sequenced, and its sequence was compared to the 23S rRNA genes of *M. genitalium* and *M. pneumoniae* (*13**,**14*).

A total of 6 bacterial species were detected in the samples (Table 1). *M. genitalium* was detected in the vaginal swab samples from 21 FSWs (14.1%, 95% CI 8.5%–19.7%). *M. genitalium* was also detected in a throat washing sample from 1 FSW (0.7%, 95% CI 0%–2.0%), whose vaginal swab sample was negative for *M. genitalium*. The sequence of the PCR product amplified from the DNA from the throat washing specimens aligned with that of the 23S rRNA gene of *M. genitalium* but not with that of *M. pneumoniae*.

**Table 1:** Bacterial species detected in vaginal swab and throat washing samples from 149 female sex workers, Japan, August 2013–January 2014

Bacterial species	No. (%) specimens positive	
	Vaginal swab	Throat washing
*Neisseria gonorrhoeae*	7 (4.7)	10 (6.7)
*Chlamydia trachomatis*	26 (17.4)	11 (7.4)
*Mycoplasma genitalium*	21 (14.1)	1 (0.7)
*Mycoplasma hominis*	52 (34.9)	2 (1.5)
*Ureaplasma parvum*	109 (73.2)	4 (2.7)
*Ureaplasma urealyticum*	52 (34.9)	5 (3.4)

The prevalence of *M. genitalium* in vaginal swab samples from the asymptomatic FSWs in this study was similar to that reported in FSWs worldwide (*7**–**10*). The FSWs enrolled in this study were at high risk for pharyngeal STIs. However, *M. genitalium* was found in only 1 throat washing sample and was not detected in throat washing samples obtained from 403 FSWs in our previous study (*11*). The prevalence of *M. genitalium* in the genitalia of FSWs would be expected to be high, whereas the prevalence of mycoplasma in the pharynx has been extremely low.

For the 21 vaginal swab samples and 1 throat washing sample that were positive for *M. genitalium* from the 22 FSWs, the portion of the 23S rRNA gene, including A-2058 and A-2059 in the 23S rRNA gene of *Escherichia coli*, and the region corresponding to the quinolone resistance–determining regions of the *E. coli gyrA* and *parC* genes were amplified from the stored DNA specimens by PCR, and sequencing of the PCR products was performed, as reported previously (*4*). The 6 FSWs to whom antibiotics had been administered 3 months before this study were not included in these 22 FSWs. 

Table 2 shows the results of analyses of these 22 specimens for the drug resistance–associated alterations. Five vaginal swab specimens and the 1 throat washing specimen could not be analyzed because their stored DNA yielded no reliable PCR products. The storage of the frozen specimens likely had degraded the quality of the DNA, or the specimens might have contained low bacterial loads of *M. genitalium*. However, for samples for which genes could be analyzed, 8 (47.1%, 95% CI 23.4%–70.8%) of 17 vaginal swab samples had macrolide resistance–associated 23S rRNA mutations, and 7 (36.8%, 95% CI 15.1%–58.5%) of 19 samples had fluoroquinolone resistance–associated *parC* alterations. For 21 vaginal swab samples, no fluoroquinolone resistance–associated *gyrA* alterations were found. Four of 16 vaginal swab samples that could be analyzed for the 23S rRNA *gyrA* and the *parC* genes showed drug resistance–associated alterations in both genes (25.0%, 95% CI 3.8%–46.2%).

**Table 2:** Mutations in the 23S rRNA gene and amino acid changes in GyrA and ParC in *Mycoplasma genitalium* detected in female sex workers, Japan*

Female sex worker	Specimen type	Mutations in the 23S rRNA gene	Amino acid changes in	
			GyrA	ParC
1	Vaginal swab	A-2058→G	–	–
2	Vaginal swab	NA	–	–
3	Vaginal swab	–	–	–
4	Vaginal swab	–	–	–
5	Vaginal swab	A-2058→G	–	–
6	Vaginal swab	A-2059→G	–	Ser-80→Ile
7	Vaginal swab	NA	–	NA
8	Vaginal swab	–	–	–
9	Vaginal swab	–	–	–
10	Vaginal swab	–	–	–
11	Throat washing	NA	NA	NA
12	Vaginal swab	–	–	–
13	Vaginal swab	NA	–	–
14	Vaginal swab	A-2059→G	–	Ser-80→Ile
15	Vaginal swab	A-2058→G	–	Ser-80→Asn
16	Vaginal swab	NA	–	Ser-80→Ile
17	Vaginal swab	–	–	Ser-80→Ile
18	Vaginal swab	–	–	–
19	Vaginal swab	–	–	Ser-80→Asn
20	Vaginal swab	A-2058→G	–	Ser-80→Asn
21	Vaginal swab	A-2058→G	–	–
22	Vaginal swab	A-2058→G	–	NA

*The nucleotide positions in the 23S rRNA gene and the amino acid positions in ParC are numbered according to *Escherichia coli* numbering. –, no mutations in the 23S rRNA gene nor amino acid changes in GyrA or ParC were found in the analyzed regions; NA, specimens could not be analyzed for the 23S rRNA, *gyrA*, or *parC* genes.

In Australia and the United Kingdom, the proportions of *M. genitalium* harboring macrolide resistance–associated mutations in clinical specimens from male and female patients with *M. genitalium* infections ranged from 36.1% to 43.4% (*2**,**3**,**5*), but proportions of the mycoplasma harboring the fluoroquinolone resistance–associated amino acid changes in *gyrA* or *parC* ranged from 4.5% to 15.4% (*2**,**3*). For Japan, we reported that drug resistance–associated 23S rRNA mutations and *parC* alterations were observed in 5 (29.4%) and 8 (47.1%), respectively, of 17 first-voided urine specimens from men with *M. genitalium*–positive nongonococcal urethritis in 2013 (*4*). The present study suggests that macrolide- and fluoroquinolone-resistant strains of *M. genitalium* will be emerging and spreading in asymptomatic FSWs and other patients with *M. genitalium* infections in Japan.

## Conclusions

This study has several limitations: the small number of enrolled FSWs, the inability to analyze all specimens for drug resistance–associated mutations, the lack of knowledge of most participants’ HIV serologic status, and the lack of longitudinal observations for FSWs with *M. genitalium* infections. Nevertheless, this study suggests that, in addition to the high prevalence of *M. genitalium* in FSWs, the mycoplasmas might frequently harbor macrolide or fluoroquinolone resistance–associated alterations. Several studies have suggested that *M. genitalium* might increase the risk for HIV acquisition in FSWs (*15*). This growing evidence indicates that *M. genitalium* infections should be included in STI control strategies for FSWs.
